# A Geometric Model to Determine Patient-Specific Cup Anteversion Based on Pelvic Motion in Total Hip Arthroplasty

**DOI:** 10.1155/2019/4780280

**Published:** 2019-05-02

**Authors:** E. Grant Sutter, Samuel S. Wellman, Michael P. Bolognesi, Thorsten M. Seyler

**Affiliations:** Department of Orthopaedic Surgery, Duke University Medical Center, Durham, NC, USA

## Abstract

**Introduction:**

Cup position is critical to stability in total hip arthroplasty and is affected by pelvis motion during positions of daily life. The purpose of this study was to explicitly define the relationship between sagittal pelvic motion and resultant cup functional anteversion and create a tool to guide the surgeon to a patient-specific intra-operative anteversion.

**Materials and Methods:**

10,560 combinations of inclination, anteversion, and pelvic tilt were generated using a geometric model. Resultant functional anteversion was calculated for each iteration and variables were correlated. An electronic mobile tool was created that compares inputted patient-specific values to population-based averages to determine pelvic positions and dynamics that may lead to instability.

**Results:**

A third-degree polynomial equation was used to describe the relationship between variables. The freely downloadable mobile tool uses input from pre-operative plain radiographic measurements to provide the surgeon a quantitative correction to intra-operative cup anteversion based on differences in functional anteversion compared to population-based averages.

**Conclusion:**

This study provides a geometric relationship between planned cup position, pelvic position and motion, and the resultant functional anteversion. This mathematical model was applied to an electronic tool that seeks to determine an individualized intra-operative cup anteversion based on measured patient-specific pelvic dynamics.

## 1. Introduction

Total hip arthroplasty (THA) is one of the most common and successful surgeries in medicine, and an accelerating number are performed each year [1]. However, instability remains a major concern, with an estimated 1% dislocation rate in primary surgery and up to 25% in revision surgery [2]. It is the most common cause of revision THA surgery, accounting for 17.3-33% of revision THA indications [1, 3, 4]. There are many factors contributing to implant stability, but component positioning is arguably the most important [5, 6]. The classic acetabular cup position “safe zone” is 40±10° inclination and 15±10° anteversion and was presented by Lewinnek et al. in 1978 [7]. It has been used widely as a baseline for appropriate cup position, though subsequent studies have demonstrated that placement of the cup within this zone does not result in reduction of dislocation rate [8–10]. One study found that in a cohort of patients who dislocated, the cup position was within this safe zone in 60% of patients, compared to 79% of patients who did not dislocate [11].

One proposed reason for this discrepancy is the concept of functional anteversion (FA), which is the resultant cup anteversion during dynamic pelvic positions such as standing and sitting [12–15]. The pelvis acts as an intercalary segment between the spine and lower extremities and rotates during dynamic movements to maintain sagittal balance and avoid bony impingement while providing hip joint stability through its relationship to the femur [16]. Because the implanted cup remains static within the acetabulum, pelvic posture changes during functional positions will uniquely alter cup orientation in space and in relation to the femoral component. This may lead to component impingement, instability, and/or accelerated wear. Previous studies have sought to determine the association between change in pelvic orientation and cup position between standing and sitting. Methods used have included plain radiographic and computed tomography (CT) imaging [17, 18], physical modeling [13, 14], and computer modeling [19–21]. The results of these studies have introduced quantitative relationships. However, these relationships are either simplified linear equations valid over small ranges or are limited by lack of granularity, making them difficult to interpret and apply clinically. Currently, there is a paucity of data providing a continuous quantitative relationship valid over a large range of cup and pelvic positions. Moreover, some previous studies have used the anterior pelvic plane (APP) [22] as the reference coordinate system rather than the global reference system, which may be more appropriate to assess the dynamic orientation of the pelvis and cup in space.

To the best of our knowledge, there is no study to date that has presented a method to directly calculate FA using an initial cup position and patient-specific sagittal pelvis motion in the global coordinate system. Therefore, the purpose of our study was multifold and included: (1) generate a quantitative relationship between a patient's planned cup position and pelvic motion from standing to sitting and the resultant FA and (2) create an electronic tool that can be used clinically in conjunction with plain radiographic measurements during pre-operative planning to help determine a patient-specific anteversion that can be applied to intra-operative cup placement.

## 2. Methods

Institutional review board approval was obtained prior to initiation of this study. Following informed consent, 24 subjects without history of inflammatory arthritis, spondyloarthropathies, surgical spinal fusion or neuromuscular disorders underwent lateral standing and sitting radiography of the lumbar spine, pelvis, and proximal femora. Pelvic tilt, sacral slope, and lumbar lordosis were measured as described previously [17, 23]. Increased positive pelvic tilt was defined as increased posterior tilt or pelvic extension i.e. sitting position will result in a higher pelvic tilt compared to standing with normal pelvic motion. Statistical differences were assessed with a paired t-test.

**Figure 1:**
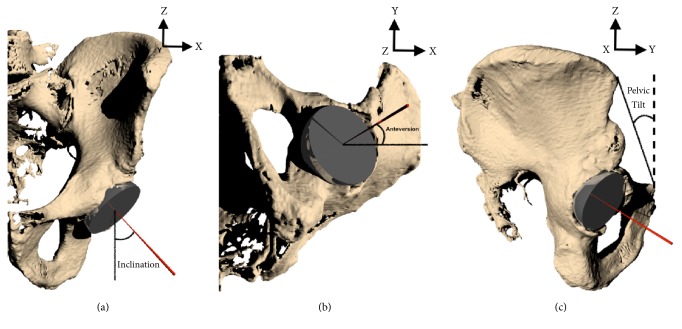
Transformations of vector normal to cup flat surface (red line) starting with (a) inclination, then (b) anteversion, and finally (c) pelvic tilt (dotted line is starting orientation and solid line is rotated orientation, with positive tilt being posterior pelvic tilt).

**Figure 2:**
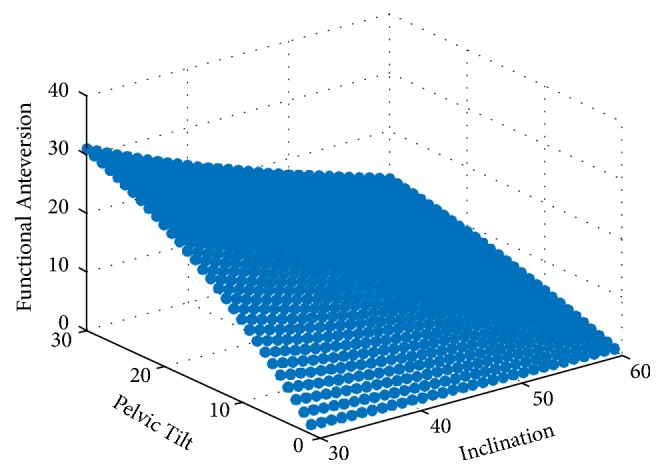
Example relationship generated to calculated change in functional anteversion based on inclination, pelvic tilt, and in this graphical example with anteversion held constant at 20°.

**Figure 3:**
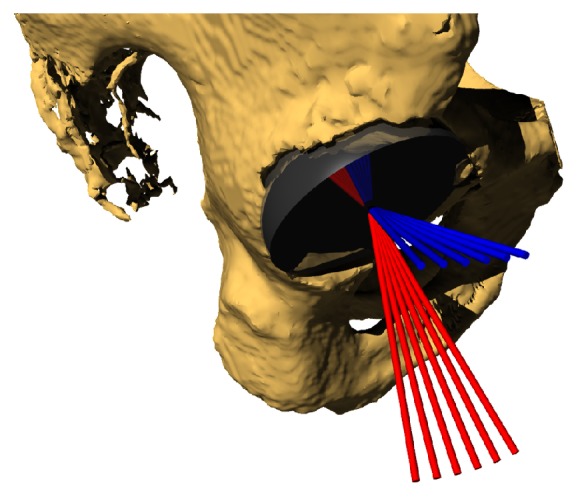
Solid modeling of pelvis, cup and vectors generated by changing pelvic tilt (rotation in sagittal plane). Red lines = vectors normal to the flat cup surface. Blue lines = red lines projected onto the axial plane representing functional anteversion.

Using numerical computing software (MATLAB, MathWorks, Inc. Natick, MA), a vector-based model was generated in a global coordinate system representing a line normal to the flat plane of an acetabular cup component. Initial cup position was based on neutral pelvic tilt, that is, the initial position of the pelvis was such that the APP was parallel to the coronal plane. The vector was then transformed within this system by adding inclination (rotation in the x-z plane, Figure 1(a)), anteversion (rotation in the x-y plane, Figure 1(b)), and pelvic tilt (rotation in the y-z plane, Figure 1(c)). The vector was then projected onto the axial plane, representing the resultant anteversion. The angle between this vector and the projected vector prior to pelvic tilt was calculated as the change in FA. Iterations of this method were performed at 1° increments from inclination 30-60°, anteversion 15-25°, and pelvic tilt 0-30°, with 10,560 resultant data points.  Figure 2 is an example of FA as a function of inclination and change in pelvic tilt when holding planned anteversion constant at 20°. Geometric model accuracy was validated using solid modeling software (Rhinoceros, Robert McNeel and Associates, Seattle, WA). A cup was modeled within a pelvis model and was manually manipulated through random transformations in the same manner as described above and FA was calculated (Figure 3). These values were then compared to the output from the geometric model and results were identical. A polynomial multivariate regression was performed in MATLAB to correlate independent variables planned inclination, planned anteversion, and change in pelvic tilt with the dependent variable being change in FA. Multiple regressions with varying polynomial orders were tested to determine the best fit and minimize error.

The mobile application was designed and developed by the authors for iOS (Apple Inc., Cupertino, CA) software using Xcode (Apple Inc.). Data inputs variables for the application are: (a) planned intra-operative cup inclination, (b) planned anteversion, (c) pelvic tilt when standing, and (d) pelvic tilt sitting obtained using radiographic measurements. Using the geometric relationship, the tool calculates the patient's FA when sitting and standing and the change in anteversion from standing to sitting. In addition, population-based pelvic tilt averages are generated using the same equation with inputted inclination and anteversion. Average population values were calculated by combining the data obtained in the current study and prior published data (Table 1) [14, 17, 24–26]. Weighted averages were calculated based on the number of subjects in each study. The average standing pelvic tilt was 0.3°; average sitting pelvic tilt was 25.8°; and average change in pelvic tilt was 25.4°.

Within the tool, patient-specific pelvic tilt when standing, sitting, and the change between standing and sitting are compared to the population-based averages. Eight scenarios of pelvic position and mobility are explored and are addressed with respective clinical recommendations (Table 2). In the case of very limited pelvic mobility, adjustments to cup anteversion may cause unpredictable effects on stability, and additional articulation options (such as dual mobility, constrained liners, or large femoral heads) are recommended. The quantitative adjustment is based on the difference between the concerning patient-specific and population-based FAs, and final output is the suggested new intra-operative cup anteversion target for surgical implantation.

## 3. Results

**Figure 4:**
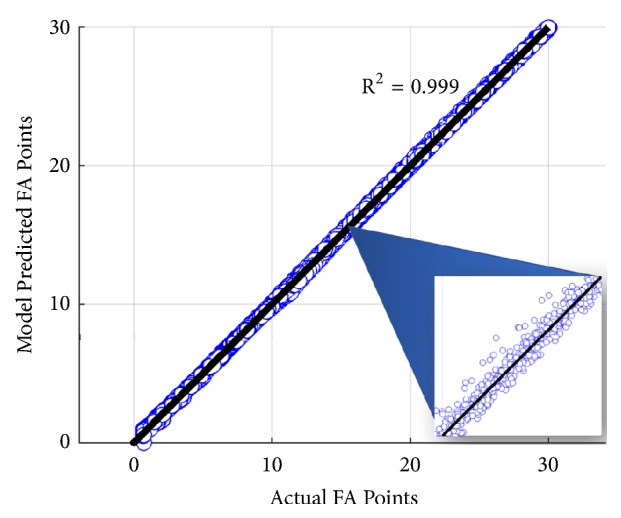
Plot of calculated functional anteversion based on the geometric model versus functional anteversion generated by the regression model equation (blue dots). Using 10,560 points, the equation was generated and fits the data (black line) with R^2^ = 0.999.

Pelvic parameters measured in this study are summarized in Table 3. To determine the geometric relationship, a third-degree polynomial regression was fit to the generated data and found to provide the best fit with R^2^ = 0.999 and average 0.06° error between modeled and calculated values (Figure 4). The change in FA can be calculated with the following equation:

(1) 3.3245·PT−0.029675·PT2+1.4579·A−0.028543·A·PT−0.000039528·A·PT2−0.065747·A2+0.000088289·A2·PT−0.62941·I−0.053399·I·PT+0.00042065·I·PT2−0.0043096·I·A+0.0003293·I·A·PT+0.00019878·I·A2+0.012728·I2+0.0001499·I2·PT−0.00004212·I2·A+0.69986−0.000070867·I3+0.00090945·A3+0.000041864·PT3

where* PT* = difference in pelvic tilt,* I* = planned inclination, and* A* = planned anteversion.

**Figure 5:**
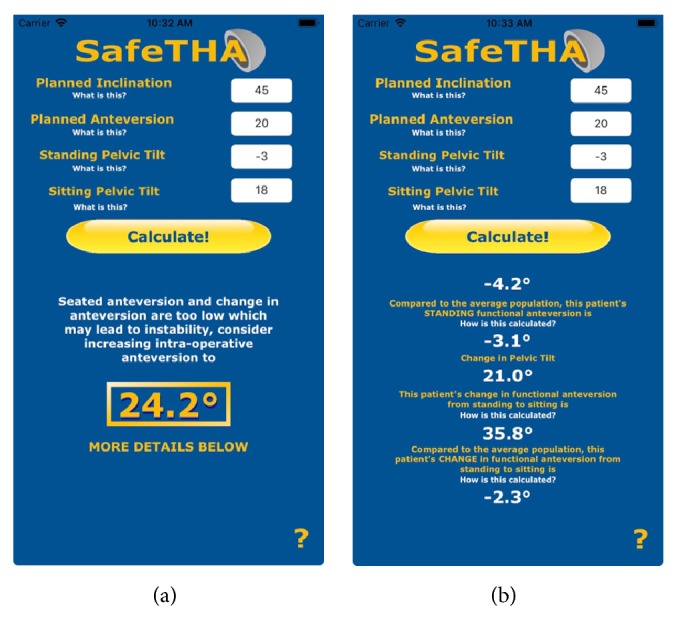
Screenshots of the “*SafeTHA*” app for Apple™ iOS mobile software that provides the user with calculated functional anteversions based on inputted desired planned inclination and anteversion, patient-specific measured standing, sitting, and change in, pelvic tilt using the mathematical relationship presented in this study. The average population-based functional anteversions are also calculated with the same planned cup position and equation. (a) The final output (boxed yellow number) is the resultant recommended new intra-operative anteversion based on these calculations and the presented algorithm. (b) Additional specifics regarding patient's anteversions compared to averages are also presented.

The resultant mobile tool is called* SafeTHA* and incorporates the equation, patient-specific and population-based values, and decision algorithm. Output is final suggested intra-operative anteversion with explanation and further details on the patient's FA when sitting, standing, and change from standing to sitting compared to the average population (Figure 5).

## 4. Discussion

The significance of dynamic pelvic parameters has been established in the literature and is increasingly considered in clinical practice. The current study presents a quantitative relationship describing cup FA from planned cup inclination, anteversion and pelvic tilt when standing, sitting, and change between the two positions. We have applied this relationship to create a mobile tool for the surgeon during pre-operative planning to help define a new, patient-specific, anteversion that seeks to reduce the risk of instability. This data can be derived from pre-operative standing and sitting plain lateral radiographs and does not require expensive advanced imaging that may also expose the patient to high doses of radiation.

Posterior instability is a risk in the setting of inadequate sitting anteversion and pelvic immobility from standing to sitting. However, this cannot be considered in isolation, and while it is tempting to consistently increase intra-operative anteversion to avoid potential posterior instability when sitting, excessive anteversion can lead to posterior impingement when standing. Our tool seeks to address this interplay and accounts for potential abnormalities throughout the arc of pelvic motion. Similarly, the studies included in the population-based average calculations reported pelvic tilt when standing and sitting, not only magnitude of change between the positions (Table 1). This full description of pelvic orientation is critical to adequately determine at-risk positions and dynamics.

When considering an appropriate population-based standard, there is a normal physiologic range of pelvic mechanics and one could consider a liberal baseline, such as the extremes of ranges measured or based on a subgroup within a standard deviation. In the current study, we chose to use averages for multiple reasons. As these are cross-sectional studies, it is likely that some portion of the population sampled had pathologic pelvic mechanics that are more likely represented at these extremes. It is also difficult to compare standard deviations across studies with varied numbers of subjects and ranges of measurements may vary within the subtleties of radiographic technique. Moreover, there is no guarantee that measurement data will fall into a normal distribution. From a clinical perspective, it is not clear at this time what values (or combination of values) portend a higher risk of instability. However, we believe that aiming for the known mean of the range provides the best opportunity to capture normal pelvic mechanics.

Previous investigations have sought to calculate a FA based on cup and pelvis position using various techniques. Kanawade et al. used a physical phantom model with set pelvic and cup parameters to determine a surrogate measure of anteversion that could be correlated with radiographic measurements [14]. Lazennec et al. used a CT-plane-modifying technique with imaging of post-operative THAs to determine that, with an average change in pelvic tilt of 30°, the average change in FA was 17.3° [18]. This study also presented an equation to determine cup anteversion, however the variables required were post-operative radiographic measurements, limiting the utility of the equation during pre-operative planning. Elkins et al. performed a finite element analysis of various cup and stem positions to determine an ideal “landing zone” of cup position based on cup diameter and stem anteversion [27]. The model is thorough and can be readily clinically applied, but does not account for a patient's unique pelvic mobility, which we believe is critical to provide a patient-specific quantitative anteversion correction.

Other studies have related inclination angle and pelvic tilt to FA. Wan et al. [21] used computer modeling and found that anteversion increased 0.7° for every increase in 1° pelvic tilt. Maratt et al. also estimated that a 1° degree change in pelvic tilt resulted in 0.74° change in FA [19]. We believe that, based on the results of our geometric model, these equations are an over-simplification of this relationship, limited to small ranges of pelvic tilt and do not account for variation in inclination or starting anteversion. Ranawat et al. similarly found a 0.75° increase in FA for every degree of pelvic tilt, but acknowledged that the relationship was accurate for inclination angles limited to 40-45° [26].

Some studies have related FA to larger ranges of discrete values of inclination, starting anteversion and pelvic tilt. However, calculations performed in these studies generated different FA values than those of the current study. For example, Marrat et al. [19] found that 40° inclination, 20° starting anteversion, pelvic and change in pelvic tilt of 15° resulted in FA of 31.1°. In contrast, with the same position, our calculations result in a FA of 34.2°. Malik et al. [20] determined that inclination 35°, anteversion 20°, and 6° of pelvic tilt from standing to sitting resulted in a change in FA of 31.3°, whereas our calculations produce a FA of 27.5°. This discrepancy is due to the difference in the coordinate reference system used to make geometric measurements. Specifically, Maratt et al. and Malik et al. measured angles in the coordinate system defined by the APP [22]. Using the APP is important to define cup placement in relation to the bony anatomy of the pelvis and for successful computer navigation or robotic-assisted surgery to ensure accurate component positioning. However, the APP is defined by the pelvis and does not remain constant in the global coordinate system [28]. We believe that it is more appropriate to use a global reference system to assess the orientation of the pelvis and cup when accounting for dynamic motion and positions in space. Referencing from the APP does account for these dynamic changes and so additional adjustments like those presented in the current study need to be combined with anatomic-based references during intra-operative cup placement [29]. The global reference system is also more applicable when taking into account cup placement with respect to the femoral component and when assessing combined anteversion [30].

Our study is the first to provide a full relationship between pelvic parameters and resultant cup orientation. However, this can be expanded to additional parameters in the dynamic chain, such as lumbar and femoral motion. Esposito et al. showed strong positive correlation between lumbar lordosis and sacral slope in both standing (R^2^ = 0.65) and sitting (R^2^ = 0.75) positions [23]. Moreover, lumbar and pelvic motion are known to decrease in symptomatic spinal deformity, degenerative spine disease, and lumbar arthrodesis [23, 31–34]. Spinal correction following THA has also shown to reduce anteversion when standing and the change in anteversion to the sitting position [33, 35]. This may account for the increased rate of instability in patients with sagittal deformity and after lumbar fusion [12, 36, 37]. The current study focuses on decreased pelvic mobility, which may be a result of altered spine mechanics, but does not elucidate these effects directly. Future studies should seek to quantitate the consequences of lumbar mobility on instability and to identify which patients may be at risk. In addition, an obligate increase in hip flexion angle when transitioning to the seated position due to loss of lumbar motion has also been demonstrated [23]. High hip flexion is a known high-risk position following THA that can lead to anterior impingement and exacerbate inadequate functional cup anteversion contributing to posterior instability [38, 39].

This study has several limitations. First, it is a modelling study using population-based measurements as standards and has not been validated clinically. However, this study provides a full geometric relationship based on variables that can be known and controlled prior to surgery and, therefore, provides a tool that can help guide pre-operative component position planning. We hope that future work will assess the clinical application of this tool and its effects on instability rate and on specific populations that may be at increased risk for dislocation. Given the relatively low event rate of instability in modern THA, this will require a large multi-center study.

We sought to address FA during positions of daily life based on patient mechanics, not pelvic position in the operating room. The surgeon cannot use this tool in a vacuum and must still understand the pelvic position intra-operatively to accurately place the cup in the desired position. We acknowledge that this tool is a simple guide to help “find the target” but that it needs to be combined with methods to “hit the target” whether via anatomic references [29, 40], computer-aided navigation [41], robotic-guided instrumentation [42], or specialized jigs [43]. Currently this method focuses on FA as it relates to posterior instability or impingement, primarily associated with the posterior approach. However, we believe that this is an appropriate starting point as posterior instability can occur in high-risk positions following both anterior and posterior approaches [44]. Moreover, the posterior approach accounts for most THAs performed throughout the world [45].

This study is the first to present a quantitative relationship and an inexpensive method to provide guidance in cup positioning to minimize instability. We believe that this method will aid the surgeon in cup position planning and may ultimately help define a new safe zone for those patients with abnormal spinopelvic motion. Further work is needed to validate this method clinically and to incorporate intra-operative tools to accurately position the component in the desired orientation. We encourage surgeons to download the free mobile application and incorporate it in their preoperative planning routine.

## Figures and Tables

**Table 1:** Data from the current study and previously studies publishing average and standard deviations of standing and sitting pelvic tilt and either reported or calculated change in pelvic tilt. Overall population-based averages were calculated from the weighted averages of these studies. NR = not recorded.

				Standing Pelvic Tilt (°)				Sitting Pelvic Tilt (°)				Change in Pelvic Tilt (°)	
Study	Year	Number of Subjects	Percentage of Total Patients	Average	Minimum	Maximum	Standard Deviation	Average	Minimum	Maximum	Standard Deviation	Average	Standard Deviation
DiGioa et al. [24]	2006	84	22	-1.2	-27	22.5	8.2	35.9	9	58	12.6	37.1	NR
Phillipot et al. [25]	2009	67	18	4.41	NR	NR	6.68	25.5	NR	NR	9.6	21.1	NR
Lazennec et al. [17]	2011	50	13	2.4	-18	20	7.3	16.9	0	44	9.1	14.5	NR
Kanawade et al. [14]	2014	85	22	-0.4	-26	15	7.4	28.3	48	5	9.1	28.7	8.6
Ranawat et al. [26]	2016	68	18	-3.7	-25	9	8.8	17.7	-7	38	11.6	21.4	12.5
Current Study	2019	24	7	4.2	25.6	-8.3	7.7	23.4	41	5.1	10.6	19.3	9.9

**Table 2:** Clinical scenarios comparing a patient's calculated standing, sitting, and change in functional anteversion to the population-based averages and the respective guidance provided by the mobile application.

Standing Anteversion	Sitting Anteversion	Change in Anteversion	Clinical Outcome	Tool Guidance
*Greater than average*	Appropriate	Appropriate	Potential for posterior impingement when standing	Decrease intra-operative anteversion

*Greater than average*	*Less than average*	Appropriate	Unlikely scenario as appropriate pelvic mobility should provide adequate seated anteversion when standing anteversion is increased	This patient has good pelvic mobility and adjustments may cause instability or impingement, maintain anteversion

Appropriate	*Less than average*	Appropriate	Low sitting anteversion may lead to posterior instability when sitting	Increase intra-operative anteversion

Appropriate	*Less than average*	*Less than average*	Low sitting and change in anteversion may lead to posterior instability when sitting	Increase intra-operative anteversion by greater discrepancy

Appropriate	Appropriate	*Less than average*	Low change in anteversion may lead to posterior instability when sitting	Increase intra-operative anteversion

*Greater than average*	Appropriate	*Less than average*	Decreased pelvic mobility and potential for impingement when standing, but patient can achieve normal sitting anteversion	Decrease intra-operative anteversion (stop if decrease leads to decreased sitting anteversion below average)

*Greater than average*	*Less than average*	*Less than average*	Patient has very limited pelvic mobility	Cup anteversion adjustments could be detrimental. Consider additional articulation options

Appropriate	Appropriate	Appropriate	Adequate pelvic mobility	No changes required

**Table 3:** Age and spinopelvic measurements of the 24 subjects included in this study. *∗*Significant difference between standing and sitting pelvic tilts (p < 0.001).

	Average	Minimum	Maximum	Standard Deviation
Age (years)	65.6	48	81	7.1

Standing Pelvic Tilt (°)	4.2	-8.3	25.6	7.7
Sitting Pelvic Tilt (°)	23.4	5.1	41	10.6
Change in Pelvic Tilt (°)*∗*	19.3	1.6	37.5	9.9

Standing Sacral Slope (°)	41.7	9.8	64.3	14.9
Sitting Sacral Slope (°)	24.3	0.6	53.4	14.7
Change in Sacral Slope (°)	-17.5	-46.3	23.2	15.6

Standing Lumbar Lordosis (°)	45.5	10.6	68.4	17.0
Sitting Lumbar Lordosis (°)	32.3	3.5	67.0	17.0
Change in Lumbar Lordosis (°)	-13.2	-37.4	22.5	14.5

## Data Availability

The patient and modeling data used to support the findings of this study are included within the article.
